# High-density mapping of atrial insertion of right lateral retrograde decremental accessory pathway: 3D illustration of accessory atrioventricular conduction network

**DOI:** 10.1016/j.hrcr.2021.11.010

**Published:** 2021-11-17

**Authors:** Philippe Maury, Quentin Voglimacci-Stephanopoli, Franck Mandel, Pauline Parlier, Maxime Beneyto, Anne Rollin

**Affiliations:** ∗ Department of Cardiology, University Hospital Rangueil, Toulouse, France; † I2MC, INSERM UMR 1297, Toulouse, France; ‡ Boston Scientific, Voisins le Bretonneuxn, France

**Keywords:** Ablation, Accessory atrioventricular node, Decremental accessory pathway, High-density mapping, Mahaim fibers

## Introduction

Atriofascicular/ventricular connections (“Mahaim fibers”) can be regarded as an accessory conduction network and very exceptionally present with retrograde conduction. High-density 3D mapping of retrograde conduction in such pathways has not been reported to date.

**Key Teaching Points**

• Reciprocating tachycardia using a retrograde right lateral decremental accessory pathway (ie, “Mahaim-like” fibers) is exceptional. Cases including a purely retrograde pathway are even more exceptional.
• High-density 3D mapping may reveal areas of specific potentials at the level of the tricuspid annulus close to the atrial insertion of the accessory pathway.
• This area of specific potentials may be regarded as an accessory conduction network.

## Case report

We report the case of a 22-year-old man with hypertrophic cardiomyopathy presenting with paroxysmal narrow QRS-supraventricular tachycardia. There was no echocardiographic sign of Ebstein anomaly.

**Figure 1:**
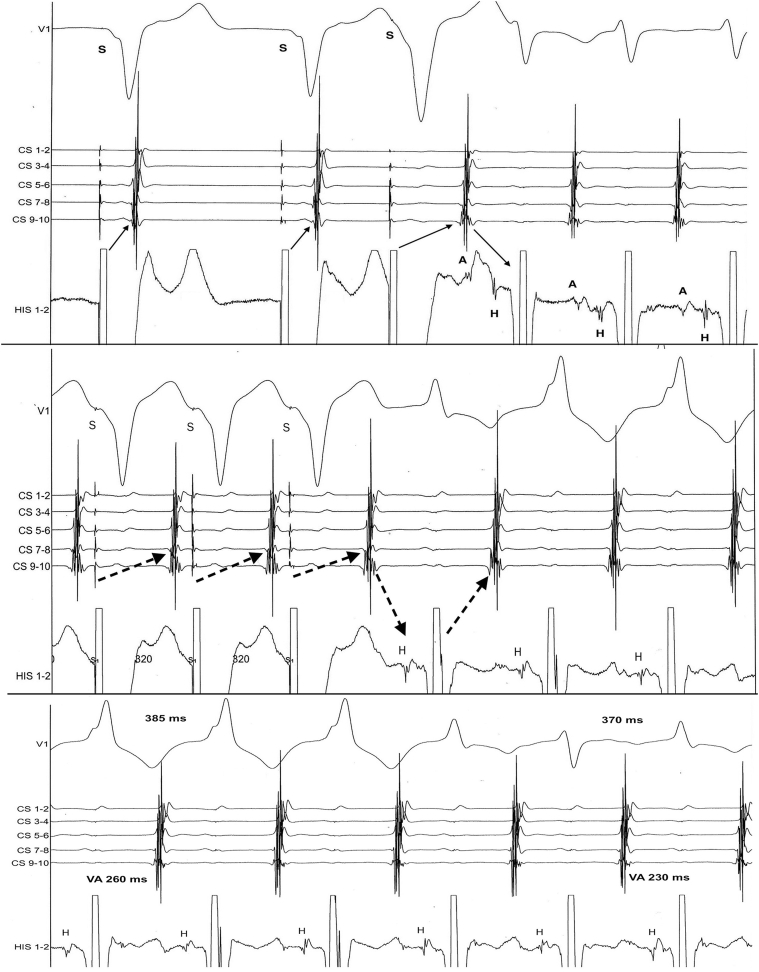
**Top:** Induction of the reciprocating tachycardia by ventricular extrastimulus with decremental retrograde conduction and a VAV pattern. **Middle:** Entrainment of the reciprocating tachycardia by fast ventricular pacing with a VAV pattern after the last ventricular paced beat, right bundle branch block, and a slower rate. Ventricular pacing could be performed from the His bundle catheter (without atrial or His capture). Postpacing interval was 445 ms at the ventricular pacing site (vs 380 ms for the tachycardia cycle length but unable to locate the circuit regarding the pacing site owing to the decremental conduction of the bypass tract). **Bottom:** Spontaneous change in tachycardia cycle length (385 to 370 ms) and VA interval (260 to 230 ms) when right bundle branch block disappeared, evoking a right lateral accessory pathway. A = atrial electrogram; CS = coronary sinus electrodes; H = His electrogram; His = His bundle electrodes; S = pacing artifact.

There was no preexcitation at baseline or during atrial pacing and no dual atrioventricular (AV) node physiology. Retrograde conduction was decremental and tachycardia was induced by ventricular extrastimulus followed by an increase in VA interval and VAV pattern (Figure 1).

**Figure 2:**
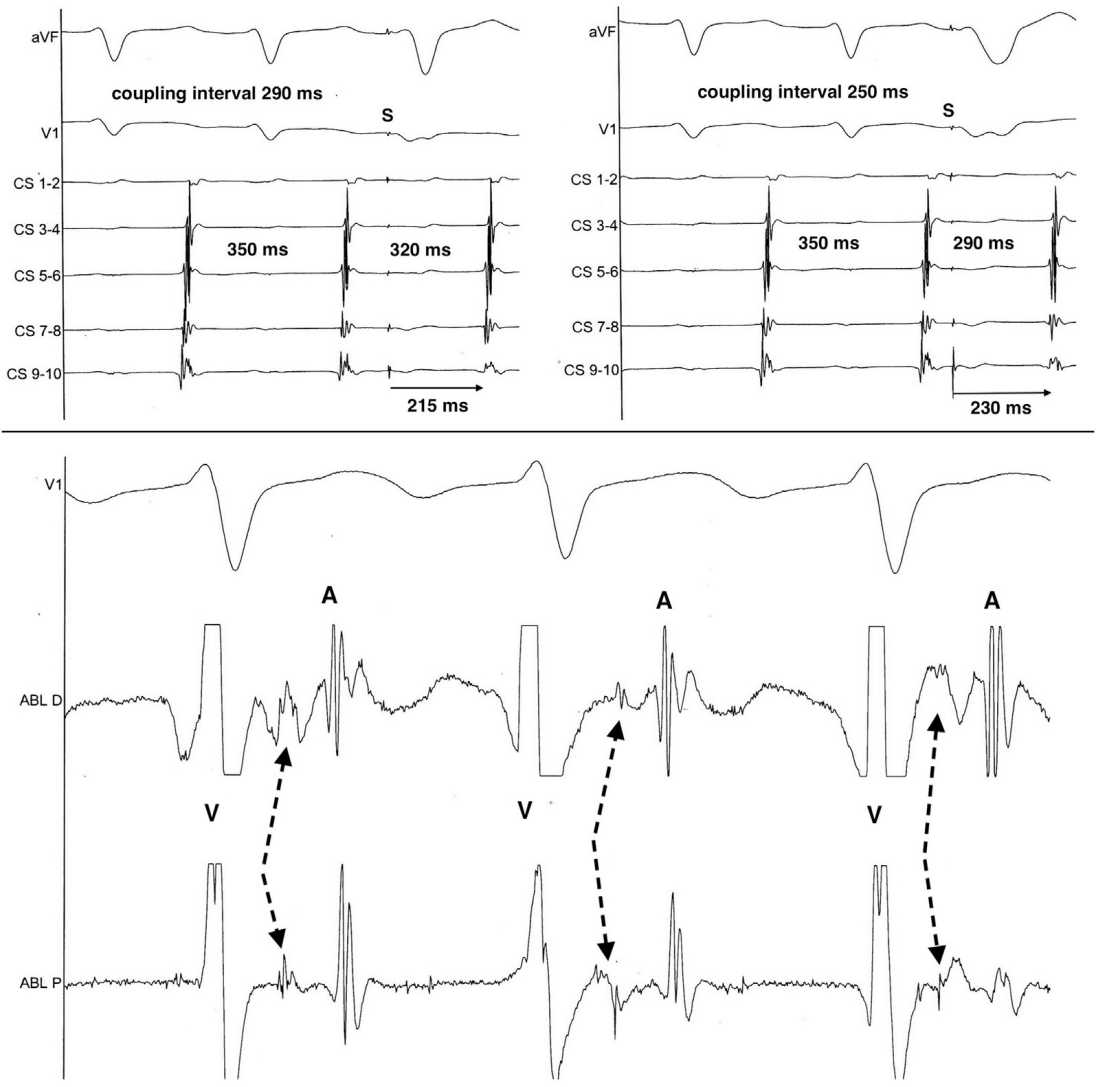
Top: Ventricular extrastimulus delivered at the time the His bundle is refractory advanced the next atrial depolarization (refractory His bundle because of pacing occurring less than 50–70 ms before theoretical His activation). Retrograde conduction through the accessory pathway has decremental properties, since although the atrial event is advanced, VA interval was larger for shorter coupling interval of the premature beat. **Bottom:** Recording by the ablation catheter at the lateral tricuspid annulus: there is a specific potential between the ventricular (V) and atrial (A) electrograms during the tachycardia, similar to a His bundle potential

Tachycardia displayed 1:1 AV relationship with relatively long VA interval (160 ms at the coronary sinus) and a VAV pattern after entrainment by ventricular fast pacing (Figure 1). Ventricular extrastimulus during tachycardia at the time the His bundle is refractory advanced the next atrial depolarization with decremental properties (Figure 2). Tachycardia rate was slower when transient right bundle branch block was present (385 vs 370 ms). Thus, reciprocating tachycardia utilizing a right bypass tract with unidirectional retrograde slow decremental conduction was diagnosed.

**Figure 3:**
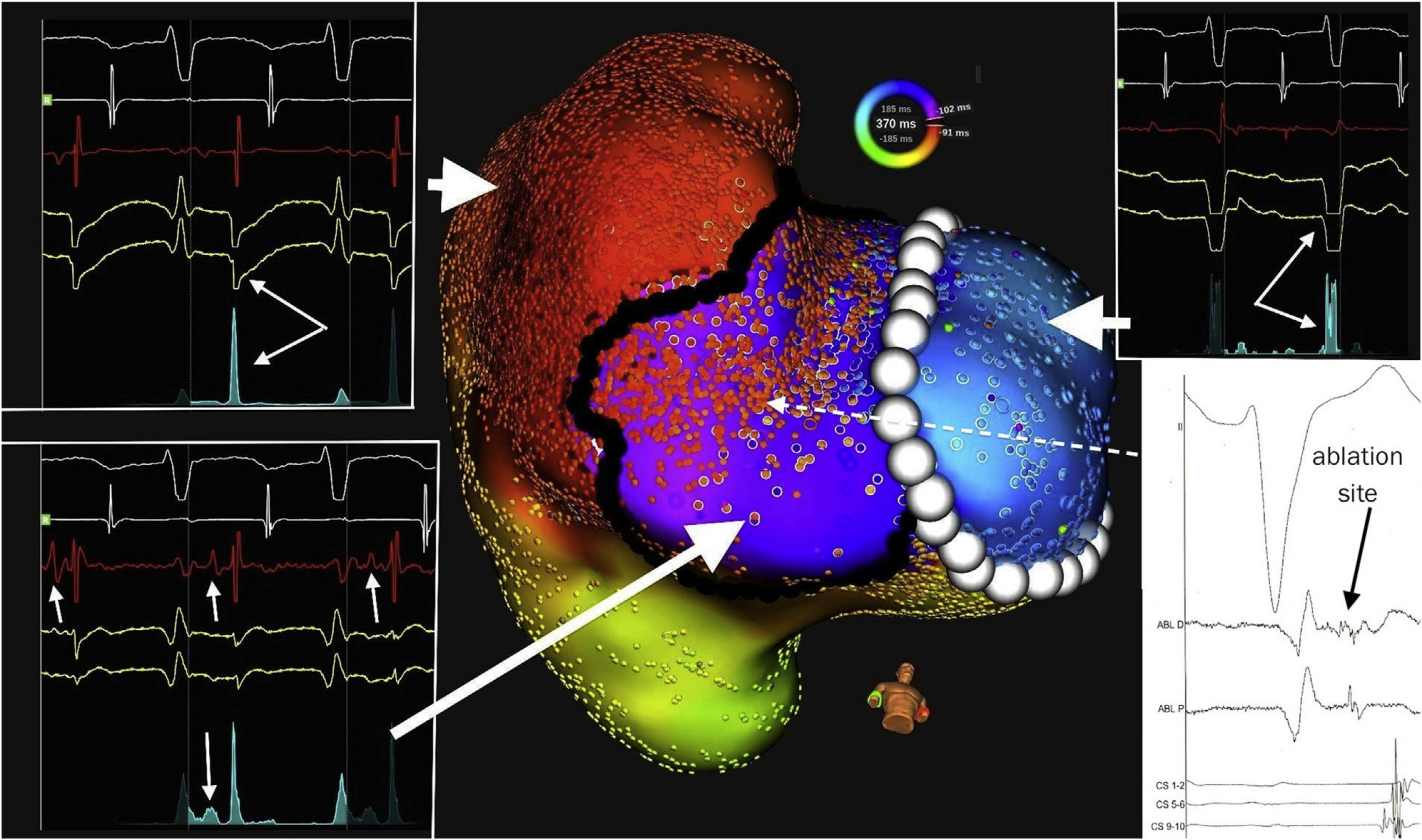
High-density 3D mapping of the right lateral part of the heart during the tachycardia, showing first the ventricular activation (*blue*) preceding the area of activation of the specific potential (*violet*) and then the atrial activation (*red and yellow*) with a concentric propagation from the preceding area. Also shown are the potentials that were recorded by the system at each site (*white* = electrocardiogram and atrial reference, *red* = local bipolar electrogram, *yellow* = local unipolar electrograms, *green* = “trend” in local activation as a summation of local electrograms). Of note, there was an additional component between the ventricular and atrial components on the trend. White dots represent the expected location of the tricuspid annulus (based on fluoroscopy and changes in local potentials) and black dots the approximate location of the additional structure responsible for the specific potential on the vestibular part of the valve, and which could be considered as an accessory atrioventricular conduction system/network. Ablation site is depicted on the lower right.

The mechanism was then more deeply investigated using an ultra-high-density 3D mapping system (Rhythmia™; Boston Scientific, Inc, Marlborough, MA). Earliest activation was mapped in the right atrium (shortest VA interval around 140 ms with large isolectrical interval), with a focal activation at the lateral part of the right atrium near the tricuspid annulus. A PPI equal to the tachycardia cycle length was elicited at this spot during atrial entrainment. Interestingly, there was a potential following ventricular activation (around 80 ms after) in a relatively large area closer to the annulus, which preceded the earliest atrial activation (around 60 ms before) (Figure 2). Reannotating on this potential and then on successive atrial signals revealed a relatively wide structure at the level of the annulus, activated from the ventricle and then conducting to the atrium in a concentric fashion (Figure 3 and Supplemental Video). Ablation at the earliest atrial site did not terminate the tachycardia, but repeated ablation attempts on the expected location of this intermediate branching structure at the level of the annulus finally terminated the tachycardia, which could never be induced again. Only decremental retrograde conduction through the AV node was present at the end of the procedure. The patient remained free of any tachycardia at 6 months follow-up.

## Discussion

The tachycardia involved a right lateral concealed bypass tract with relatively long and decremental retrograde conduction and without anterograde conduction (acting like purely retrograde Mahaim fibers). This extraanatomical retrograde conduction was associated with a particular wide structure located at the tricuspid annulus, harboring specific potential, and compatible with an accessory AV node and conduction pathway. To our knowledge, this is the first 3D documentation of such a retrograde accessory pathway and of a potential accessory AV node and conduction network.

Atriofascicular/ventricular connections (Mahaim fibers) usually do not demonstrate retrograde conduction^1^^,^^2^ and are considered to represent an accessory AV conduction pathway^2^ because of decremental properties and because of a specific potential—similar to His potential—recorded at the tricuspid annulus and further along the right lateral ventricular free wall.^1^^,^^2^ It is expected to consist of a proximal component (similar to the AV node) at or above the tricuspid annulus, and a distal component (similar to the His bundle) that generates the specific potential, extending toward the right ventricular apex and distal components of the right bundle branch.^2^ Accessory AV node has been identified as an insulated tract of specialized cardiomyocytes, piercing the insulating pathways of the atrioventricular junction and extending into the right ventricle, thus producing a second AV conduction system located on the lateral part of the tricuspid annulus, representing remnants of AV ring tissue.^3^ Atrial components of decremental accessory pathways, found in the vestibule of the tricuspid valve, could harbor remnants of the tissues that give rise to the normal AV node.^4^ Localization of the area of recording of a specific potential in our case was consistent with the location of the vestibule of the tricuspid valve^4^ and thus could appear to be a 3D representation of what has been described in autopsies.^3^^,^^4^ The relative wide area of recording was not indicative of common accessory pathway potentials, nor was the long VA interval evocative of conduction through an usual accessory pathway where local ventricular and atrial events are commonly fused. We were not able to depict the proximal (ventricular) end of the accessory pathway, since there was no anterograde conduction.

Three-dimensional mapping of decremental AP has been published, but only for anterograde conduction.^5^, ^6^, ^7^ Rarely, such decremental atriofascicular/ventricular connections demonstrated retrograde conduction.^8^, ^9^, ^10^ Concealed retrograde conduction had been demonstrated through the distal part of a decremental accessory pathway that blocked at its proximal atrial insertion (specific potential not followed by atrial activity).^11^ Orthodromic reciprocating tachycardia utilizing such pathways has sometimes been documented,^9^^,^^10^^,^^12^ but only in patients with anterograde conduction and preexcitation. Thus, it seems to be the first report of orthodromic reciprocating tachycardia using a purely concealed decremental accessory pathway with findings compatible with an accessory AV node.

Some more common bypass tracts, however, may also have decremental properties without being categorized as Mahaim fibers: these “short decrementally conducting AV pathways” may simply be due to alteration of conduction properties or tortuosity.^4^^,^^11^ However they usually do not present with retrograde conduction^2^^,^^12^ and large areas of specific potentials are not described in this setting. Therefore, this case may be considered as a 3D illustration of orthodromic tachycardia involving an accessory AV node at the level of the tricuspid annulus in a patient without preexcitation.

## Appendix Supplementary data

Supplemental Video 3D activation movie showing the retrograde activation through the right lateral accessory pathway, with an area of specific potentials at the level of the tricuspid annulus, which can be regarded as an accessory conduction network.
